# Eosinophils, basophils and myeloid-derived suppressor cells in chronic *Loa loa* infection and its treatment in an endemic setting

**DOI:** 10.1371/journal.pntd.0012203

**Published:** 2024-05-21

**Authors:** Gerrit Burger, Rafiou Adamou, Ruth Kreuzmair, Wilfrid Ndzebe Ndoumba, Dorothea Ekoka Mbassi, Anne Marie Nkoma Mouima, Carole Mamgno Tabopda, Roukoyath Moyoriola Adegnika, Ayong More, Dearie Glory Okwu, Lia-Betty Dimessa Mbadinga, Carlos Lamsfus Calle, Luzia Veletzky, Wolfram Gottfried Metzger, Benjamin Mordmüller, Michael Ramharter, Ghyslain Mombo-Ngoma, Ayola Akim Adegnika, Rella Zoleko-Manego, Matthew B. B. McCall

**Affiliations:** 1 Centre de Recherches Médicales de Lambaréné (CERMEL), Lambaréné, Gabon; 2 Institute of Tropical Medicine, University of Tübingen, Tübingen, Germany; 3 Department of Implementation Research, Bernhard Nocht Institute for Tropical Medicine & I Department of Medicine, University Medical Center Hamburg-Eppendorf, Hamburg, Germany; 4 German Center for Infection Research, Partner sites Hamburg-Borstel-Lübeck-Riems, Germany; 5 Centre for Tropical Medicine, Bernhard Nocht Institute for Tropical Medicine & I Department of Medicine, University Medical Center Hamburg-Eppendorf, Hamburg, Germany; 6 Department of Medicine I, Division of Infectious Diseases and Tropical Medicine, Medical University of Vienna, Vienna, Austria; 7 Department of Internal Medicine IV, University of Tübingen, Tübingen, Germany; 8 Department of Medical Microbiology, Radboud University Medical Center, Nijmegen, The Netherlands; 9 German Center for Infection Research, Partner site Tübingen, Tübingen Germany; NIAID-ICER, INDIA

## Abstract

**Background:**

Chronic infection by *Loa loa* remains an unsolved immunological paradox. Despite harboring subcutaneously migrating adult worms and often high densities of microfilariae, most patients experience only relatively mild symptoms, yet microfilaricidal treatment can trigger life-threatening inflammation. Here, we investigated innate cell populations hypothesized to play a role in these two faces of the disease, in an endemic population in Gabon.

**Methodology/Principal findings:**

We analyzed numbers and activation of eosinophils and basophils, as well as myeloid-derived suppressor cell (MDSC) subsets and associated circulating cytokine levels by flow cytometry in sex- and age-matched *L*. *loa*-uninfected (LL-), -amicrofilaraemic (MF-) and -microfilaraemic (MF+) individuals (n = 42), as well as microfilaraemic individuals treated with albendazole (n = 26). The percentage of eosinophils was lower in LL- (3.0%) than in the combined *L*. *loa*-infected population, but was similar in MF+ (13.1%) and MF- (12.3%). Upon treatment of MF+, eosinophilia increased from day 0 (17.2%) to day 14 (24.8%) and had decreased below baseline at day 168 (6.3%). Expression of the eosinophil activation marker CD123 followed the same pattern as the percentage of eosinophils, while the inverse was observed for CD193 and to some extent CD125. Circulating IL-5 levels after treatment followed the same pattern as eosinophil dynamics. Basophil numbers did not differ between infection states but increased after treatment of MF+. We did not observe differences in MDSC numbers between infection states or upon treatment.

**Conclusions/Significance:**

We demonstrate that both chronic infection and treatment of *L*. *loa* microfilaraemia are associated with eosinophil circulation and distinct phenotypical activation markers that might contribute to inflammatory pathways in this setting. In this first ever investigation into MDSC in *L*. *loa* infection, we found no evidence for their increased presence in chronic loiasis, suggesting that immunomodulation by *L*. *loa* is induced through other pathways.

## Author summary

Loiasis–also called African eye worm for its most pathognomonic feature–is a disease caused by the helminth *Loa loa*, affecting over ten million people in Central and West Africa. While *L*. *loa* seems to successfully evade an effective immune response in chronic infection, treatment can lead to severe inflammation upon rapid death of *L*. *loa* larvae in the blood. Here, we investigated different subsets of immune cells hypothesized to play a role in *L*. *loa*-mediated immune modulation in chronically infected individuals and upon treatment of individuals with circulating larvae. We found that eosinophils–an important cell population in the immune response to helminths–increase in numbers and are activated in both *L*. *loa*-infection and its treatment. Myeloid-derived suppressor cells can suppress immune responses in cancer and some other infectious diseases but have never been investigated in loiasis. We hypothesized that they are involved in immunosuppression by *L*. *loa* but found no evidence thereof. Better understanding of immunomodulation in chronic loiasis may ultimately improve the management of this neglected disease.

## Introduction

Loiasis is a filarial disease caused by the nematode *Loa loa*. Also known as African eye worm for its pathognomonic manifestation of adult worms migrating through the conjunctiva, loiasis is endemic in Central and West Africa [1,2], where at least 10 million people are infected [3]. Until recently, loiasis was primarily considered of public health importance due to severe adverse events that occur in *L*. *loa*-infected individuals receiving treatment in the context of mass drug administration programs against onchocerciasis and lymphatic filariasis in areas of co-endemicity. The drugs used in these programs–ivermectin and diethylcarbamazine (DEC)–also have a strong microfilaricidal effect on *L*. *loa* microfilariae. An association between severe adverse inflammatory reactions and *L*. *loa* microfilaraemia was first established in Cameroon, where it was estimated that individuals harboring more than 30,000 *L*. *loa* microfilariae per milliliter of blood (mf/ml) are at significant risk of serious neurological reactions following ivermectin treatment [4–7]. However, loiasis deserves attention as a major public health issue in its own right: a recent cross-sectional burden of disease study conducted in rural Gabon found a prevalence of over 70% in some communities and a disease burden estimated at 412.9 disability-adjusted life years (DALYs) per 100,000 inhabitants [8]. In addition, microfilaraemic individuals have a higher risk of mortality [9,10]. Although it is evident that loiasis causes significant morbidity and mortality in affected populations, it remains so neglected that it does not even feature on the WHO’s formalized list of neglected tropical diseases, in spite of urgent appeals to address this issue [2,11,12].

The clinical spectrum of *L*. *loa*-infection is characterized by a remarkable dichotomy. Acute incidental infection in short-term visitors to endemic regions generally presents with manifest symptoms and a pro-inflammatory state typified by profound hypereosinophilia, episodes of angioedema and hypergammaglobulinemia [13]. In contrast, chronic infection in life-long residents of endemic regions seems to be more diverse in clinical outcome. While it has often been described as relatively asymptomatic, more recent data suggest a spectrum of more frequent albeit non-specific symptoms associated with chronic loiasis despite high burdens of infection [8,14]. Interestingly, microfilaraemic individuals experience *fewer* Calabar swellings and *less* pruritus than amicrofilaraemic individuals (i.e. infection involving only adult worms) [15,16]. *L*. *loa* has moreover been associated with higher frequency of other filarial [17] and human T-lymphotropic virus (HTLV) [18] infections, as well as impaired CD4+ memory T cell responses to tuberculosis antigen [19], suggesting a state of immunosuppression that extends to bystander-antigens. However, once microfilaraemic individuals are treated with microfilaricidal drugs such as ivermectin and DEC, they risk developing life-threatening inflammatory reactions. Although treatment with albendazole is usually not associated with acute immunological reactions, similar severe adverse events have been reported [20–22].

The complex interplay between parasite and host immune response that underlies these paradoxical facets of chronic loiasis remains poorly understood. IL-5 driven eosinophils are the primary effector cell type thought to be responsible for parasite death and feature prominently in acute incidental infection, though their role in chronic infection and treatment-driven adverse events is less clear [23–27]. Basophils have emerged as important contributors to the immune response against parasitic [28,29] and filarial [30,31] infection by initiating and amplifying type 2 cytokine production. However, they have never been investigated in loiasis. On the other hand, in order for adult worms to survive for 10 years or even longer whilst migrating subcutaneously, they must evade, modulate and/or suppress the host’s immune defenses–a characteristic common amongst many other parasites [32–36]. Of particular interest in this regard are myeloid-derived suppressor cells (MDSC), a heterogeneous population of immature immune cells capable of abrogating natural killer, B and T cell responses. First and most extensively described for their role in facilitating immune-evasion in cancer, their immunomodulatory activity is also known to be exploited by pathogens including various helminths and other parasites [37–43]. However, their role in loiasis has previously never been investigated.

The present study aimed to further improve our understanding of the enigmatic bipolar immunology of chronic loiasis by investigating innate cell populations in different infection states and following treatment of *L*. *loa*.

## Methods

### Ethics statement

Written informed consent was obtained from each study participant or a legal representative. The study was conducted in accordance with the Declaration of Helsinki, ICH-GCP guidelines and local regulations. The immunological study was approved by the CERMEL (Centre de Recherches Médicales de Lambaréné) Institutional Ethics Committee (reference number CEI-022/2018), as were the underlying cross-sectional study (CEI-011/2017) and treatment trial (CEI-013/2017).

### Study design and population

This immunological study was conducted in the area surrounding Lambaréné, Gabon between March 2018 and December 2020. Loiasis, other helminth infections and malaria are highly prevalent in this rural setting [15,44,45]. Immunological analysis was performed on a subset of participants enrolled in two ongoing studies: a large cross-sectional burden of disease study [8] and an open-label randomized controlled trial assessing the efficacy of different albendazole-based treatment regimens in 42 *L*. *loa*-microfilaraemic individuals [46].

Forty-two malaria-RDT-negative (Paracheck Pf, Orchid Biomedical Systems, Goa, India) individuals of at least two years of age were included in a case-control design from among participants enrolled in the cross-sectional burden of disease study. Participants were selected pragmatically by best match across infection status by sex, age, and region. They were matched by sex and age across three groups by *L*. *loa* infection status: (i) microfilaraemic (detectable *L*. *loa* microfilaraemia), (ii) amicrofilaraemic (history of eye worm, no detectable *L*. *loa* microfilaraemia, detectable anti-*L*. *loa* IgG) and (iii) uninfected (no history of eye worm, no detectable *L*. *loa* microfilaraemia, no detectable anti-*L*. *loa* IgG).

In the randomized controlled trial (Pan African Clinical Trials Registry ID PACTR201807197019027), microfilaraemic adult individuals (who had not received albendazole treatment during the four previous weeks) were allocated randomly at a 1:2:2:2 ratio to receive no causal treatment, 3 weeks of albendazole (800 mg/d), 5 weeks of albendazole or 3 weeks albendazole + sequential single-dose ivermectin (150 μg/kg). Gastrointestinal helminth infections were assessed at baseline by stool microscopy in most participants, but were not significantly associated with the immunological parameters (e.g., eosinophil numbers, Tables B and C in S1 Appendix). Because all participants received the same treatment until sampling for immunology at day 14 and no significant differences in immunological responses between treatment regimens were observed at the end of follow-up (day 168), a pooled analysis combining the three treatment regimens was performed in our immunological study. Thus, all twenty-six participants who received one of the albendazole-based regimens and completed follow-up were available for immunological analysis.

### *Loa loa* diagnosis

History of eye worm was evaluated by the rapid assessment procedure for *L*. *loa* (RAPLOA) questionnaire [47]. *Loa loa* microfilaraemia was assessed by microscopy of venous EDTA blood collected between 10:00 and 15:00 hours because *L*. *loa* has diurnal periodicity of microfilaraemia. Microscopy was performed on thick blood smears or after leukoconcentration, as appropriate. Participants were considered amicrofilaraemic only after applying leukoconcentration technique with saponin lysis and microscopy of 1 ml of blood, as described previously [8]. Co-infection with *Mansonella perstans* (*M*. *perstans*) was documented. *Loa loa* serology was performed on EDTA plasma using the *L*. *loa* SXP-1 IgG rapid lateral flow immunoassay (Drugs & Diagnostics for Tropical Diseases, San Diego, USA) according to the manufacturer’s instructions. Results were obtained visually, and any detectable line was considered positive.

### Immunology

A 10 ml tube of sodium heparin anticoagulated venous blood was collected for immunological assays once from each participant in the cross-sectional study and at baseline (day 0), during albendazole treatment (day 14) and at the end of follow-up (day 168) from each participant in the treatment trial. All cellular flow cytometry was performed on a Guava EasyCyte 8 HT flow cytometer using Guava InCyte version 2.7 (Merck Millipore, Burlington, USA). Flow cytometry data were analyzed with FlowJo version 10.6.1 (BD Biosciences, San Jose, USA). Clones and isotypes of all antibodies used for flow cytometry are listed in Table A in S1 Appendix.

### Eosinophils and basophils

For eosinophil and basophil characterization, whole blood was incubated with anti-human CD125 (IL5Ra, interleukin 5 receptor alpha) PE, CD193 (CCR3, CC motif chemokine receptor 3) PerCP-Cy5.5 (both BD Biosciences), CD69 FITC, CD123 (IL3R, interleukin 3 receptor) PE-Cy7, CD16 (FcγRIII) APC-Cy7 and CX3CR1 (CX3C motif chemokine receptor 1) APC (all BioLegend, San Diego, USA) for 15 minutes. Erythrocyte lysis was performed with FACS lysing solution (BD Biosciences) for 10 minutes, after which samples were washed twice in phosphate-buffered saline (PBS) (Thermo Fisher Scientific, Waltham, USA). Percentages of SSC^hi^ CD125+ CD193+ eosinophils and SSC^lo^ CD123+ CD193+ basophils among single leukocytes were determined (for gating strategy, see Fig A in S1 Appendix), as described elsewhere [48,49]. Of note, during initial assay development Siglec-8 was assessed as an additional marker but was not found of added value to CD123 and CD193 in discriminating eosinophils. Expression of activation markers was recorded as median fluorescence intensity (MFI) or percentage of positive events, as appropriate. CD123 is a marker of eosinophil progenitors and upregulated upon activation, [50,51] while CD125 and CD193 are downregulated in a negative feedback loop [50,52,53]. Surface expression of CD16 and CD69 have been associated with eosinophil activation [51]. Activated basophils express less CD193 [54].

### Myeloid-derived suppressor cells (MDSC)

For MDSC quantification, peripheral blood mononuclear cells (PBMC) were isolated by density gradient centrifugation (Ficoll-Paque PLUS, GE Healthcare, Chicago, USA) according to published procedures [55]. Next, 250,000 PBMC were blocked with rabbit serum (Capricorn Scientific, Ebsdorfergrund, Germany) and stained with anti-human CD66b FITC, HLA-DR (human leukocyte antigen–DR isotype) PerCP, CD14 APC-Cy7 (all BD Biosciences), CD33 PE and CD11b (integrin alpha M) APC (both Miltenyi Biotec, Bergisch Gladbach, Germany) for 20 minutes. Freshly isolated samples were analyzed. Percentages of SSC^hi^ CD66b+ CD11b+ CD14- polymorphonuclear (PMN-‍)MDSC and SSC^lo^ CD14+ HLA-DR- CD11b+ CD33+ monocytic (M-)MDSC among single PBMC were determined (Fig A in S1 Appendix), as described elsewhere [43].

### PMN-MDSC isolation and T cell proliferation-suppression assay

As proof-of-principle–to confirm that PMN-MDSC defined phenotypically by flow cytometry are indeed functionally suppressive–a T cell proliferation-suppression assay was performed in a subset of participants (selected pragmatically by the total number of PBMC available) following a published protocol [43]. Briefly, participant PMN-MDSC were isolated by magnet-activated cell sorting (MACS), added to healthy donor PBMCs at varying effector-to-target ratios and their effect on CD4+ and CD8+ T cell proliferation assessed by flow cytometry (S2 Appendix and Fig A in S1 Appendix).

### Cytometric bead array (CBA)

Heparin plasma was separated by centrifugation, aliquoted and stored at -80°C. Cytokines were assessed using a commercial cytometric bead array (CBA) kit (BD Biosciences) as recommended by the manufacturer. Briefly, this procedure uses multiplex bead array technology to simultaneously detect IL-1b, IL-4, IL-5, IL-6, IL-10, and IFN-g, TNF-a, GM-CSF. Samples were acquired on a FACS Symphony A1 flow cytometer (BD Biosciences) using FACS Diva software and the Median Fluorescence Intensities (MFI) were generated using FlowJo version 10.7.1 software. Standard curves were generated by analyzing the serial dilutions of the standards spanning the concentration range from 1 to 2,500 pg/ml using GraphPad Prism version 6 (GraphPad Software, San Diego, USA). Lower limits of quantification were 1 pg/ml; samples with cytokine concentrations below the lower level of quantification were represented as half the value of the lower limit of quantification.

### Statistical analyses

Data analysis was performed in R version 3.5.3 (R Foundation for Statistical Computing, Vienna, Austria) within the tidyverse package version 1.3.0. [56] Categorical data were summarized by frequency and numeric data by median and interquartile range (IQR). Proliferation indices in the T cell suppression assay were normalized to the stimulated control. Friedman tests were used to assess differences between participants in the cross-sectional study and within-subject differences across time points in the treatment study. Nemenyi’s post-hoc test was applied after significant Friedman results. Spearman rank analysis was used for correlation between immunological parameters and microfilaraemia. P-values < 0.05 were considered statistically significant.

## Results

### Study population

We included 33 female and 9 male participants in the cross-sectional study, with a median age of 25 years (Table 1). Age was similar between *L*. *loa*-microfilaraemic (MF+), amicrofilaraemic (MF-) and uninfected (LL-) participants (p = 0.61). Co-infection with *M*. *perstans* was present in only one MF- and two MF+ participants. In the clinical trial, we included 13 female and 13 male participants who received an albendazole-based treatment regimen and completed immunological sampling and analysis. Microfilaraemia at enrolment (median: 9,000; IQR: 7,500–15,000 mf/ml) decreased to 24% of baseline (2,200; 1,100–4,500 mf/ml) and 16% of baseline (1,400; 430–5,100 mf/ml) on day 14 and day 168 after start of treatment, respectively (Fig B in S1 Appendix). Microfilaraemia with *M*. *perstans* was detected by thick blood smear in 8 of 26 individuals at baseline. *M*. *perstans* microfilaraemia was below 500 mf/ml at baseline and became undetectable upon albendazole treatment in these 8 participants. Stool microscopy for gastrointestinal helminths at baseline was performed in 18 of 26 (69%) participants and yielded positive results in 7 of 18 (39%) participants (Tables 1 and B in S1 Appendix). Complete eosinophil/basophil and MDSC data were each available for in total 22 participants.

**Table 1:** Study population.

Characteristic	Uninfected (n = 14)	Cross-sectional Amicrofilaraemic (n = 14)	Microfilaraemic (n = 14)	Clinical trial Treated (n = 26)
**Sex, no. female (% total)**	11 (79)	11 (79)	11 (79)	13 (50)
**Median age, years (IQR)**	28 (16–42)	24 (16–44)	24 (15–47)	61 (47–72)
**Median *Loa loa*, mf/ml (IQR)**	-	-	26 (3–150)	9,000 (7,500–15,000)
***M*. *perstans +*, no. (% total)**	0 (0)	1 (7)	2 (14)	8 (13)
**GI helminths +, no. (% total)**	-	-	-	7/18 (39)*

* Stool microscopy was performed in 18 of 26 participants in the treatment study.

### Eosinophils

**Fig 1:**
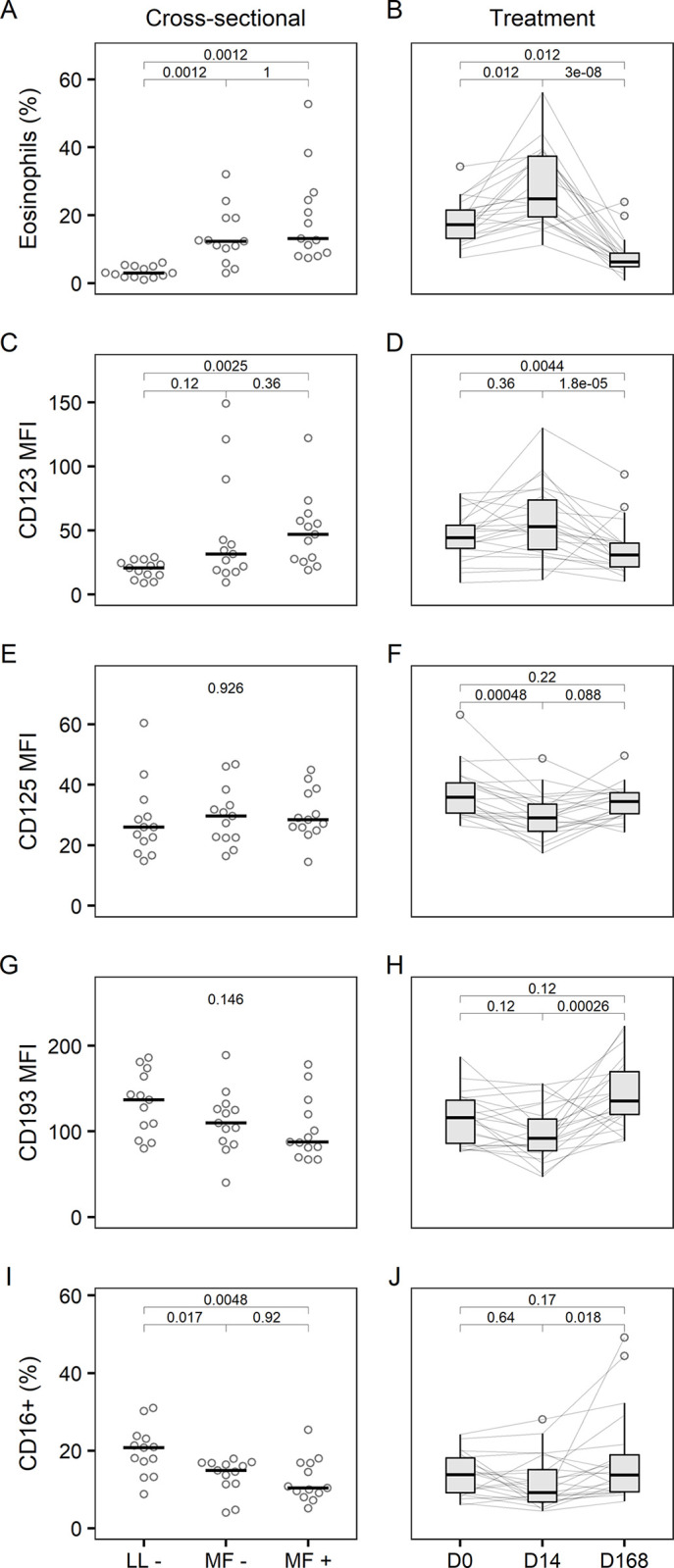
Eosinophil numbers and surface activation markers. Percentage of SSC^hi^ CD125+ CD193+ eosinophils among single leukocytes and expression of surface activation markers as depicted by median fluorescence intensity (MFI) or percentage positive in 39 age- and sex-matched *Loa loa*-uninfected (LL-), -amicrofilaraemic (MF-) and -microfilaraemic (MF+) participants in the cross-sectional study (left column, horizontal bars represent median) and in 22 microfilaraemic participants receiving different albendazole-based treatment regimens at baseline (D0), on day 14 of treatment (D14) and at the end of follow-up (D168) (right column, Tukey’s boxplot gives median, interquartile range (IQR) and whiskers 1.5 IQR). P values were obtained by Friedman’s and Nemenyi’s post-hoc test. Matched eosinophil data were unavailable for three participants in the cross-sectional and four participants in the treatment study.

Eosinophilia, as a percentage of total leukocytes, did not differ significantly between microfilaraemic (MF+, median: 13.1%; IQR: 9.0–24.5) and amicrofilaraemic (MF-, 12.3%; 10.2–19.2) *L*. *loa*-infected participants in the cross-sectional study, but was low in uninfected (LL-, 3.0%; 1.8–5.0) participants (Fig 1A). In the treatment study, eosinophilia increased from 17.2% (13.2–21.5) at baseline to 24.8% (19.5–37.4) at day 14 post-treatment and subsequently decreased to below baseline at day 168 (6.3%; 4.9–8.9, Fig 1B). Moreover, CD123 expression (median fluorescence intensity–MFI), a marker of young eosinophils and their progenitors, was higher in MF+ (46.9; 27.7–57.4) and to some extent in MF- (31.6; 18.9–42.5) than in LL- participants (20.8; 15.2–24.5, Fig 1C); and upon treatment tended to increase from baseline (44.2; 36.1–54.2) to day 14 (52.9; 35.1–73.9), followed by a decrease to below baseline at day 168 (30.8; 21.7–40.2, Fig 1D). MFI of CD125–a marker that is downregulated in activated eosinophils–did not differ between groups in the cross-sectional study (MF+: 28.4, 25.9–37.1; MF-: 29.6, 22.5–33.2; LL-: 26.0, 21.3–29.4, Fig 1E). However, in contrast to eosinophil numbers and CD123 expression, CD125 MFI decreased upon treatment from baseline (35.8; 30.6–40.6) to day 14 (29.0; 24.6–33.6) and increased again to 34.4 (30.5–37.3) at day 168 (Fig 1F). CD193 MFI–that is also known to be downregulated upon eosinophil activation–was lower in MF+ (87.4; 81.1–120) than in MF- (110; 88.8–126) and lower still than in LL- participants (137; 107–164, Fig 1G), though not statistically significantly and–similarly to CD125 expression–decreased from baseline (116; 86.2–136) to day 14 (92.0; 77.4–114), followed by an increase above baseline at day 168 (136; 120–170, Fig 1H). Analogously, CD16 expression–which is considered a marker of eosinophil activation–was lower in MF+ (10.4%; 9.1–16.8) and MF- (14.9%; 11.5–16.8) than LL- (20.8%; 17.2–23.1) participants (Fig 1I), appeared to decrease slightly between baseline (13.8%; 9.2–18.1) and day 14 (9.2%; 6.8–15.1) and then increased again to around baseline levels at day 168 (13.7%; 9.5–18.9, Fig 1J). The percentage of eosinophils expressing CX3CR1 and CD69 MFI did not differ significantly between groups in the cross-sectional or between time points in the treatment study. CD16 expression correlated inversely with baseline microfilaraemia in the treatment (r = -0.43, p = 0.031), but not in the cross-sectional study (Fig C in S1 Appendix). We found no significant correlation between microfilarial density at day 0, 14 or 168, or the drop in microfilaraemia between day 0 and day 14, and other eosinophil parameters in the two data sets (Figs C and D in S1 Appendix).

### Basophils

**Fig 2:**
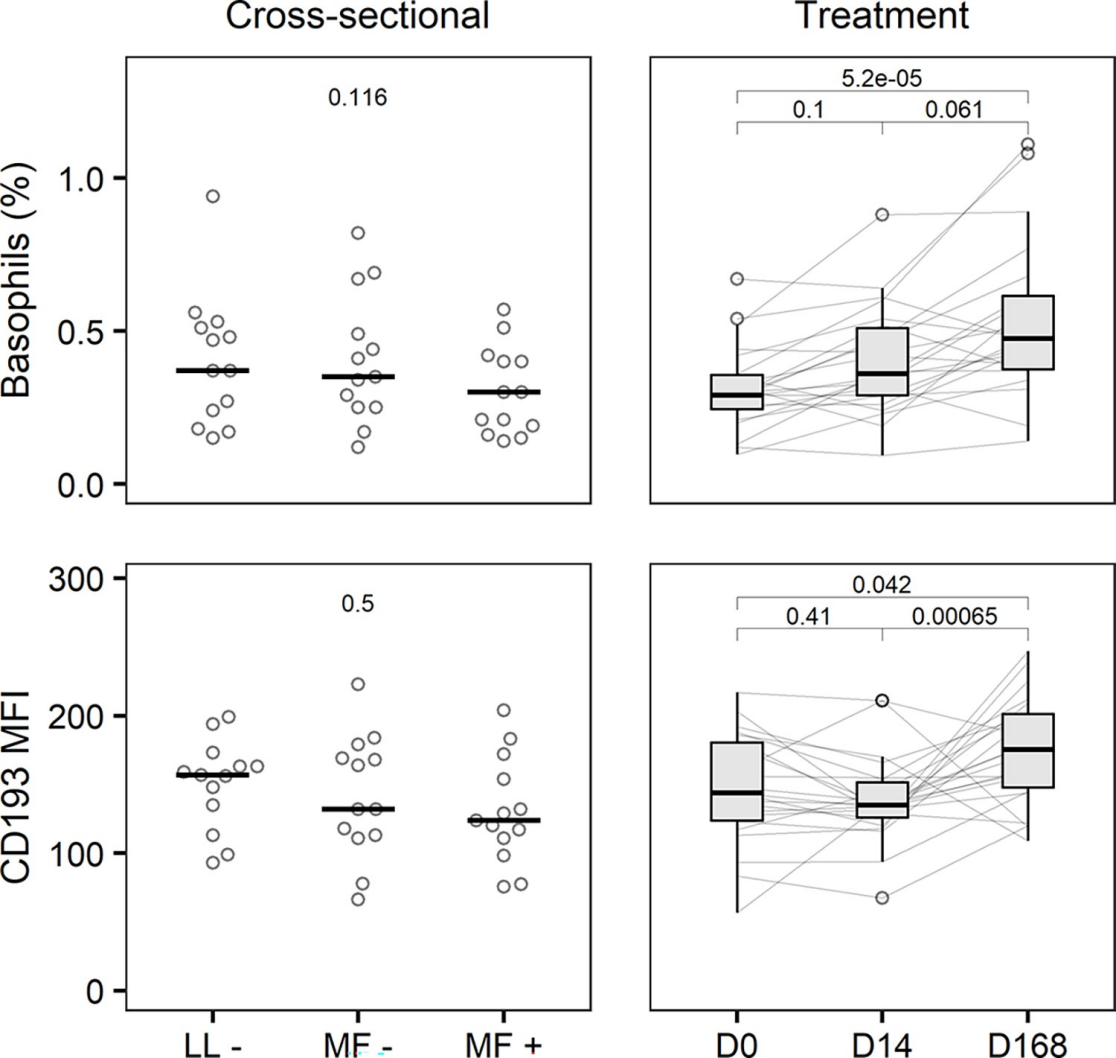
Basophil numbers and CD193 expression. Percentage of SSC^lo^ CD123+ CD193+ basophils among single leukocytes and expression of CD193 median fluorescence intensity (MFI) in 39 age- and sex-matched *Loa loa*-uninfected (LL-‍), -amicrofilaraemic (MF-) and -microfilaraemic (MF+) participants in the cross-sectional study (left column, horizontal bars represent median) and in 22 microfilaraemic participants receiving different albendazole-based treatment regimens at baseline (D0), on day 14 of treatment (D14) and at the end of follow-up (D168) (right column, Tukey’s boxplot gives median, interquartile range (IQR) and whiskers 1.5 IQR). P values were obtained by Friedman’s and Nemenyi’s post-hoc test. Matched basophil data were unavailable for three participants in the cross-sectional and four participants in the treatment study.

Basophil numbers, as a percentage of total leukocytes, did not differ between groups in the cross-sectional study (MF+: 0.30%, 0.19–0.40; MF-: 0.35%, 0.25–0.49; LL-: 0.37%, 0.24–0.51; Fig 2). However, upon treatment they tended to increase from baseline (0.29%; 0.25–0.36) to day 14 (0.36%; 0.29–0.51) to day 168 (0.48%; 0.38–0.62). We found no difference in basophil CD193 MFI between MF+ (124; 111–154), MF- (132; 113–169) and LL- (157; 135–163) participants. In the treatment study CD193 MFI remained stable between baseline (144; 124–180) and day 14 (135; 126–152) but thereafter increased to 176 (148–202) at day 168.

### Myeloid-derived suppressor cells (MDSC)

**Fig 3:**
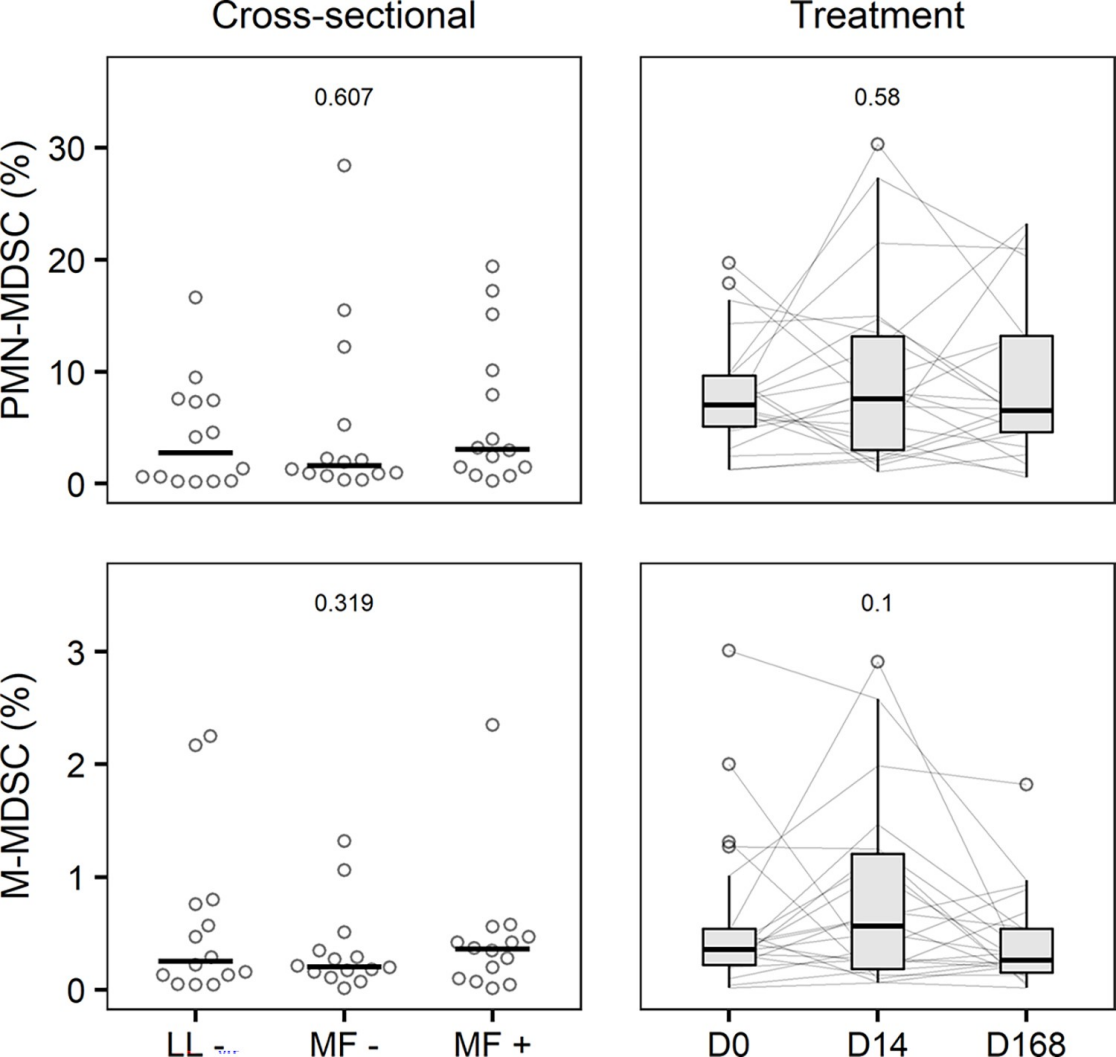
Myeloid-derived suppressor cells (MDSC). Percentage of SSC^hi^ CD66b+ CD11b+ CD14- polymorphonuclear (PMN-)MDSC and SSC^lo^ CD14+ HLA-DR- CD11b+ CD33+ monocytic (M-)MDSC among single PBMC in 42 age- and sex-matched *Loa loa*-uninfected (LL-), -amicrofilaraemic (MF-) and -microfilaraemic (MF+) participants in the cross-sectional study (left column, horizontal bars represent median) and in 22 microfilaraemic participants receiving different albendazole-based treatment regimens at baseline (D0), on day 14 of treatment (D14) and at the end of follow-up (D168) (right column, Tukey’s boxplot gives median, interquartile range (IQR) and whiskers 1.5 IQR). P values were obtained by Friedman’s and Nemenyi’s post-hoc test. Matched MDSC data were unavailable for four participants in the treatment study.

We found no difference in PMN-MDSC numbers between MF+ (3.08%; 1.46–9.56), MF- (1.60%; 0.90–4.50) and LL- (2.75%; 0.34–7.40) participants in the cross-sectional study (Fig 3). Despite high individual variation of PMN-MDSC numbers over the course of treatment, no clear trend was observed between day 0 (7.02%; 5.10–9.68), day 14 (7.58%; 3.04–13.2) and day 168 (6.53%; 4.62–13.2). There was no difference in M-MDSC numbers between MF+ (0.36%; 0.13–0.46), MF- (0.21%; 0.16–0.34) and LL- (0.26%; 0.13–0.71) participants. Although not statistically significant, we observed a trend towards an increase in M-MDSC numbers between day 0 (0.36%; 0.22–0.54) and day 14 (0.57%; 0.19–1.20), followed by a decrease back to around baseline at day 168 (0.26%; 0.15–0.54). We found no significant correlation between baseline microfilarial density or drop in microfilaraemia between day 0 and day 14 and MDSC numbers.

### T cell proliferation-suppression assay

T cell proliferation-suppression assays were performed using PMN-MDSC from a subset of five study participants in three independent experiments in combination with freshly isolated PBMC from a single healthy *L*. *loa*-uninfected donor. The purity and viability of PMN-MDSC isolated by magnet-activated cell-sorting were > 90% and > 95%, respectively. PMN-MDSC from study participants showed a dose-dependent suppression of both CD4+ and CD8+ T cell proliferation indices by 15 to 41% at the highest PMN-MDSC to PBMC ratio (Fig E in S1 Appendix). Control mature PMN granulocytes from the same participants did not show suppression of T cell proliferation.

### Circulating cytokines

**Fig 4:**
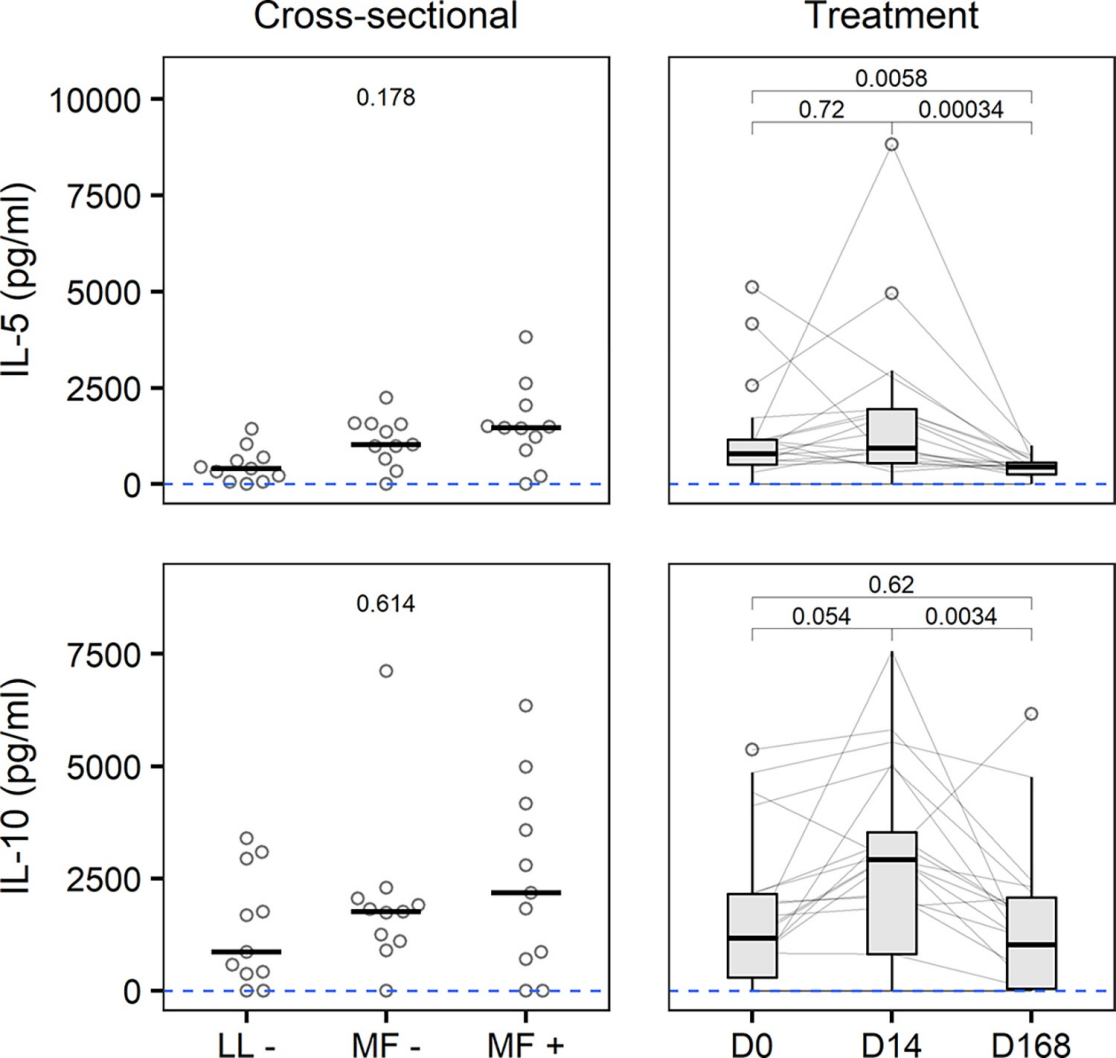
Circulating cytokines. Plasma levels of interleukin 5 (IL-5) and interleukin 10 (IL-10) in 33 age- and sex-matched *Loa loa*-uninfected (LL-), -amicrofilaraemic (MF-) and -microfilaraemic (MF+) participants in the cross-sectional study (left column, horizontal bars represent median) and in 21 microfilaraemic participants receiving different albendazole-based treatment regimens at baseline (D0), on day 14 of treatment (D14) and at the end of follow-up (D168) (right column, Tukey’s boxplot gives median, interquartile range (IQR) and whiskers 1.5 IQR). P values were obtained by Friedman’s and Nemenyi’s post-hoc test. Matched cytokine data were unavailable for nine participants in the cross-sectional and five participants in the treatment study. Three and nine IL-5 measurements and five and 15 IL-10 measurements were below the lower limit of quantification (blue dashed line, 1 pg/ml) in the cross-sectional and treatment study, respectively.

We found no statistically significant differences in levels of any of the circulating cytokines that we measured between LL-, MF- and MF+ individuals in the cross-sectional study (Figs 4 and F in S1 Appendix). Following albendazole treatment, circulating IL-5 levels followed the pattern of eosinophil dynamics, dropping to below baseline again by day 168 (D0: 780 pg/ml, 510–1160; D14: 940 pg/ml, 540–1960; D168: 450 pg/ml, 260–560; Fig 4). We also observed a trend to increased circulating IL-10 levels between day 0 (1160 pg/ml, 300–2160) and day 14 (2920 pg/ml, 820–3530), which then dropped again by day 168 (1020 pg/ml, 40–2080). No significant changes were seen in interleukin 1 (IL-1), IL-4, IL-6, granulocyte-macrophage colony-stimulating factor (GM-CSF), interferon gamma (IFNg) and tumor necrosis factor alpha (TNFa) levels, either during or following albendazole treatment (Fig F in S1 Appendix).

## Discussion

We set out with the hypothesis that chronic loiasis is associated with marked immunosuppression, whilst treatment would result in inflammatory responses particularly in the eosinophil compartment.

Indeed, we found that both eosinophilia and eosinophil activation, as well as circulating IL-5 levels, increased during treatment and dropped to below baseline six months thereafter, in line with several treatment studies in endemic [26,57,58] and non-endemic [59] populations. This inflammatory response is thought to be induced by parasite antigen released from dying microfilaria following administration of microfilaricidal drugs like ivermectin and DEC, as also suggested by recent observations in a baboon model [60]. Microfilarial counts in our treatment trial dropped somewhat faster than was observed in some but not all similar studies, which might be related to differences in dose [61] or duration [62–64] of albendazole administered, or the extensive variability in its pharmacokinetics [65]. The rapid decrease in microfilaraemia and such evident eosinophil responses following albendazole treatment suggest that this embryostatic drug may also have some microfilaricidal activity and/or that it exerts direct toxicity against adult filaria, resulting in antigen release and subsequent eosinophilic inflammation. Its effects moreover appear relatively long-lasting, as not only did microfilarial counts remain significantly lower at end of follow-up (six months after treatment), but immunological parameters had normalized beyond baseline.

In contrast with the only previous study to investigate eosinophils in *L*. *loa* infection in a cross-sectional setting in a highly endemic, Central African population, [66], but in common with some previous reports from elsewhere (including endemic residents presenting at Western travel clinics) [67,68], we also found *L*. *loa*-infection to be associated to eosinophilia, eosinophil activation and to some extent circulating IL-5 levels. Moreover, these all appeared marginally more pronounced in microfilaraemic compared to amicrofilaraemic participants, as observed again more clearly in a recent study at our research center [16]. The discrepant results of the original Gabonese study [66], may in retrospect be explained by the absence of serostatus for *L*. *loa*-specific antibodies in its case-definition (because e.g. anti-SXP-1 antibodies were not assessed), as some amicrofilaraemic infections may have been missed and erroneously included in the *L*. *loa*-negative population.

These immunological observations would fit with recently published evidence that far from being a largely benign condition, chronic loiasis is in fact associated with significant morbidity [8]. Indeed, in highly endemic regions, *L*. *loa*-infection is responsible for a sizeable loss of disability-adjusted life years (DALYs). We thus consider that even in chronically infected individuals, activated eosinophils continue to play an important role in the immunopathogenesis of this disease.

Whereas the expression of most eosinophil surface markers that we measured followed a congruent pattern, the percentage of eosinophils expressing CD16 –a marker commonly associated with eosinophil activation–was *higher* in *L*. *loa*-uninfected individuals and appeared to *decrease* further during treatment of *L*. *loa*-microfilaraemic participants, while *increasing* significantly thereafter. CD16 upregulation of eosinophils has been linked to pro-inflammatory cytokines (IFN-γ, IL-2), IgG immune complexes, states of allergy and soil-transmitted helminth infection [69–72]. However, recent work actually proposing a regulatory role for CD16+ “suppressive eosinophils” underlines that understanding of eosinophil phenotypes and functions is still in its infancy and deserves further research–particularly in loiasis [73]. Finally, these observations must be interpreted knowing that eosinophil function and activity may differ in different tissues and observations in peripheral blood do not have to reflect their function and activation in other organs.

Basophils have been shown to play an important role in the response against helminth infections [28–31], but this study is the first to assess *in vivo* basophil responses in chronic loiasis. We did not observe significant differences in either basophil percentages or basophil activation between uninfected and (microfilaraemic) infected individuals or *during* albendazole treatment. By the end of follow-up, however, basophil percentages had *increased* significantly above baseline in treated participants, yet their activity appeared to be *diminished* in comparison with baseline, as indicated by significantly higher CD193 expression. The implications thereof remain unclear.

Our study also investigated for the first time the potential role of MDSC in chronic loiasis. Although PMN-MDSC from *L*. *loa*-infected individuals are functionally suppressive, we observed no significant difference in either PMN- or M-MDSC percentages between uninfected and (a-)microfilaraemic infected individuals or in response to albendazole treatment. While the percentage of M-MDSC tended to slightly increase temporarily upon treatment, this presumably represents a physiological regulatory response to a primary inflammatory response. MDSC expansion has been linked to a variety of chronic parasitic infections [37], including a mouse model of filarial infection, where MDSC expanded in the thoracic cavity, the site of infection with *Litomosoides sigmodontis* [74]. Although we cannot exclude MDSC expansion in subcutaneous tissues in response to migrating adult *L*. *loa*, rather than in blood to circulating microfilariae, MDSC expansion in peripheral blood is common in both solid tumors [75] and tissue-dwelling parasitic infections [76]. It thus remains noteworthy that we did not find evidence of elevated MDSC numbers in chronic loiasis. However, future studies could also investigate MDSC activation and function in this setting.

Other than IL-5, the only other circulating cytokine that we were able to detect significant changes in was IL-10. In line with this, a previous study at our center noted that *L*. *loa* microfilaraemic participants harbored greater numbers of IL-10 producing CD4+ T cells than amicrofilaraemic individuals [77]. Indeed, filarial antigen can drive the production of IL-10 in infected individuals, suggesting expansion of regulatory responses [78]. Animal models of *L*. *loa* infection have shown that Th1 and Th2 responses are downregulated upon patency, leading to T cell hyporesponsiveness and potentially preventing parasite clearance [79–81]. In humans, *L*. *loa* has been associated with impaired CD4+ memory T cell responses to tuberculosis antigen [19], suggesting a state of immunosuppression that extends to bystander-antigens. However,–as our MDSC data underline–the key cellular players in *L*. *loa*-mediated immunomodulation remain to be identified. In one previous study at our center, T regulatory cells in cord blood from offspring of *L*. *loa*-infected mothers correlated inversely with Th1 and Th17 cells, which thus deserves further investigation as an immunoregulatory pathway in loiasis [82]. However, other mechanisms such as modulation of innate pattern-recognition receptor responses or of antigen presentation and co-stimulatory pathways might also contribute to immunomodulation by *L*. *loa*.

One limitation of our current study–apart from small sample size–is the relatively low microfilarial density in our cross-sectional population. Since in our endemic setting both the prevalence and burden of *L*. *loa*-infection is strongly correlated with age, our study design called for age-matching of uninfected and infected individuals and inclusion of a wide range of ages to avoid confounding due purely to immunosenescence. An unintended consequence thereof, was low median parasitemia in our matched microfilaraemic individuals, which may have limited our ability to detect specific immunological patterns associated with microfilariae as opposed to infection itself. However, we did include some highly microfilaraemic individuals (up to >40,000/ml) in the treatment study, but other than an inverse correlation with CD16 expression on eosinophils, we found no immunologic parameters correlating significantly with microfilaraemia. A second limitation is the restricted microfilaricidal activity of albendazole in comparison with drugs like ivermectin and DEC; in combination with an absence of high microfilarial densities, this may have diminished our chances of witnessing the most fulminant immune responses to dying microfilariae. Although one of the treatment arms in our study did involve follow-up administration of ivermectin, this sub-group was too small to allow meaningful immunological assessments. A general limitation of treatment studies concerning loiasis applies to our longitudinal population: There are currently no means to assess the direct impact of therapy on adult worms. Even though we observed a significant decrease in microfilaraemia, adult worms might have survived and continued to modulate the host`s immune responses–dampening the observable effect of treatment on the immune responses. Finally, we were not able to exclude all other helminth infections that could potentially interfere with the immunological observations in our study population. However, participants in the cross-sectional study were selected by best match by sex, age and region to avoid confounding by exposure. In the treatment study, gastrointestinal helminth co-infection was assessed and not associated with immunological differences. Thus, although confounding due to other unidentified helminth co-infections cannot be excluded, we mainly consider infection by *L*. *loa* to be responsible for the immunological effects reported here.

Notwithstanding our recent documentation of the significant morbidity caused by chronic loiasis [8] and our demonstration of eosinophil activation in chronically-infected individuals, it remains remarkable that *far more* inflammation does not occur in response to subcutaneously migrating adults and circulating microfilariae, given that minute quantities of allergen and bacterial toxins like lipopolysaccharide may cause anaphylaxis and life-threatening sepsis, respectively. Deeper molecular understanding of the regulatory pathways induced by this remarkable parasite to ensure its own survival so successfully may ultimately lead to improved management of this neglected disease.

## Supporting information

S1 Appendix Supplementary data. Supplementary Figures and Tables.
(PDF)

S2 Appendix Supplementary methods. T cell proliferation-suppression assay.
(PDF)

S1 File Data file cross-sectional study. (XLSX)

S2 File Data file treatment study. (XLSX)
