# Supplementary material for: Eosinophils, basophils and myeloid-derived suppressor cells in chronic Loa loa infection and its treatment in an endemic setting
Source: PLoS Negl Trop Dis. 2024 May 21;18(5):e0012203. doi: 10.1371/journal.pntd.0012203 (PMC11147522; doi:10.1371/journal.pntd.0012203)
Supplement: S1 Appendix — Supplementary Figures and Tables. (PDF) [file pntd.0012203.s001.pdf]

Supplementary data

Table A: Clones and isotypes of antibodies for flow cytometry

Target	Fluorochrome	Clone	Isotype (species)
CD4	PE	RPA-T4	IgG1 (mouse)
CD8a	APC	HIT8a	IgG1 (mouse)
CD11b	APC	M1/70.15	IgG2b (rat)
CD14	APC-Cy7	MφP9	IgG2b (mouse)
CD16	APC-Cy7	3G8	IgG1 (mouse)
CD33	PE	AC104.3E3	IgG1 (mouse)
CD66b	FITC	G10F5	IgM (mouse)
CD69	FITC	FN50	IgG1 (mouse)
CD123	PE-Cy7	6H6	IgG1 (mouse)
CD125	PE	A14	IgG1 (mouse)
CD193	PerCP-Cy5.5	5E8	IgG2b (mouse)
CX3CR1	APC	2A9-1	IgG2b (rat)
HLA-DR	PerCP	L234	IgG2a (mouse)

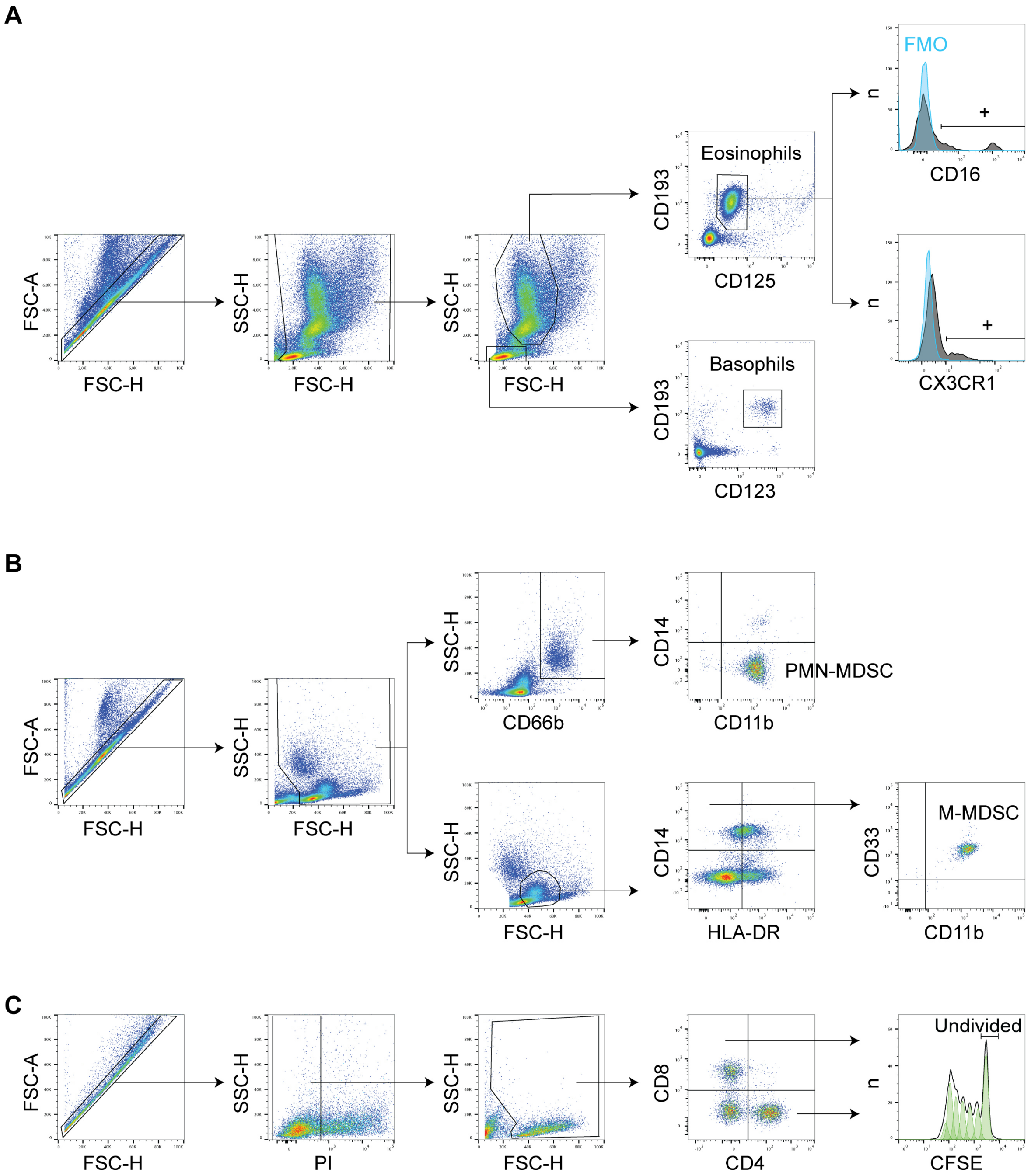

Figure A: Flow cytometry gating strategies

A: Eosinophils were defined as SSC^{hi} CD125+ CD193+ and basophils as SSC^{lo} CD123+ CD193+ among single leukocytes, respectively. Expression of activation markers was conveyed as median fluorescence intensity (MFI) or percentage of positive events (CD16 and CX3CR1 in eosinophils; FMO: fluorescence minus one control). B: Polymorphonuclear myeloid-derived suppressor cells (PMN-MDSC) were defined as SSC^{hi} CD66b+ CD11b+ CD14- and monocytic (M-)MDSC as SSC^{lo} CD14+ HLA-DR- CD11b+ CD33+ among single peripheral blood mononuclear cells (PBMC), respectively. C: Single live (propidium iodide (PI) negative) CD4+ and CD8+ PBMC were assessed for carboxyfluorescein diacetate succinimidyl ester (CFSE) expression by FlowJo Proliferation Modelling (BD Biosciences, San Jose, USA) to calculate proliferation indices (total number of cell divisions divided by cells that went into division).

Table B: Co-Infections with gastrointestinal helminths in the treatment study

	n	% (n=18)
**Stool microscopy positive**	7	39
Trichuris trichiura	4	22
**Hookworm *spp.***	3	17
Ascaris lumbricoides	1	6
Strongyloides stercoralis	1	6

Results of baseline stool microscopy in 18 out of 26 participants included in the immunological analysis of the treatment study, for whom these results were available.

Table C: Immunological outcomes by gastrointestinal helminth co-infection

	No GI helminth co-infection	GI helminth co-infection	Wilcoxon p
**n**	11	7	-
**Eosinophils, %**	17.7 (13.2-21.5)	23.8 (17.2-30.2)	0.261
**Basophils, %**	0.275 (0.218-0.353)	0.29 (0.225-0.48)	0.696
**PMN-MDSC, %**	6.73 (5.88-9.79)	7.16 (3.59-8.72)	0.786
**M-MDSC, %**	0.4 (0.21-1.16)	0.32 (0.24-0.45)	0.468

Main baseline immunological outcomes by gastrointestinal helminth co-infection in 18 out of 26 participants included in the immunological analysis of the treatment study, for whom these results were available. Gastrointestinal co-infection was defined as detection of ova of *Trichuris trichiura*, *Ascaris lumbricoides* and/or hookworm *spp.* and/or *Strongyloides stercoralis* larvae by microscopy in a participant's stool.

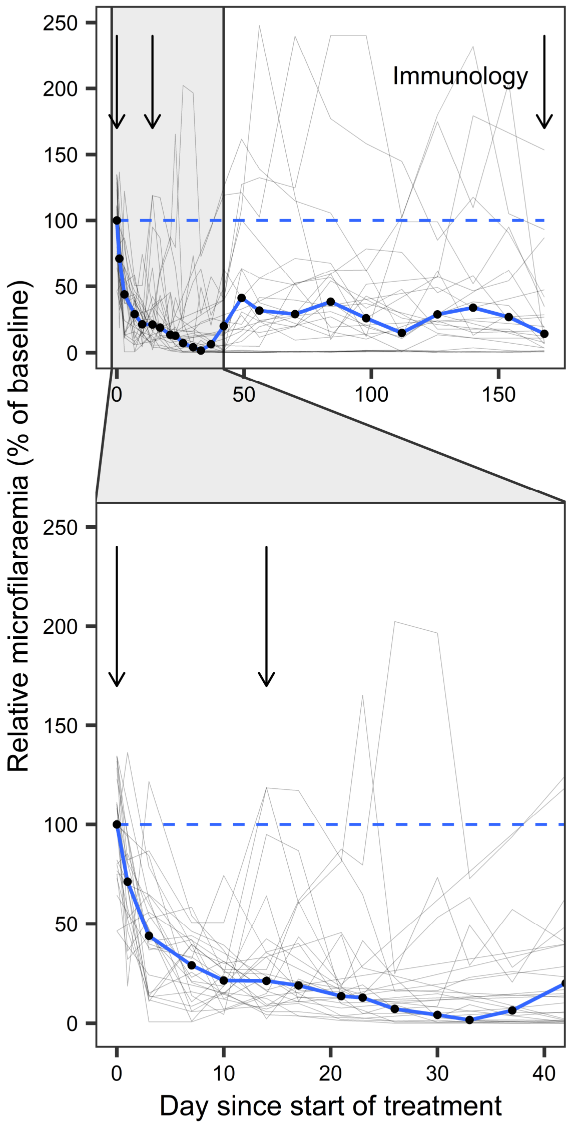

Figure B: Relative microfilaraemia of participants in the clinical trial

Relative microfilaraemia (normalized to mean of screening and baseline) of 26 microfilaraemic participants undergoing albendazole-based treatment regimens (3 weeks of albendazole, 3 + 2 weeks of albendazole or 3 weeks albendazole + single-dose ivermectin). Grey lines represent individual data, black dots the median, the dotted line the baseline microfilaraemia and the arrows sampling for immunology.

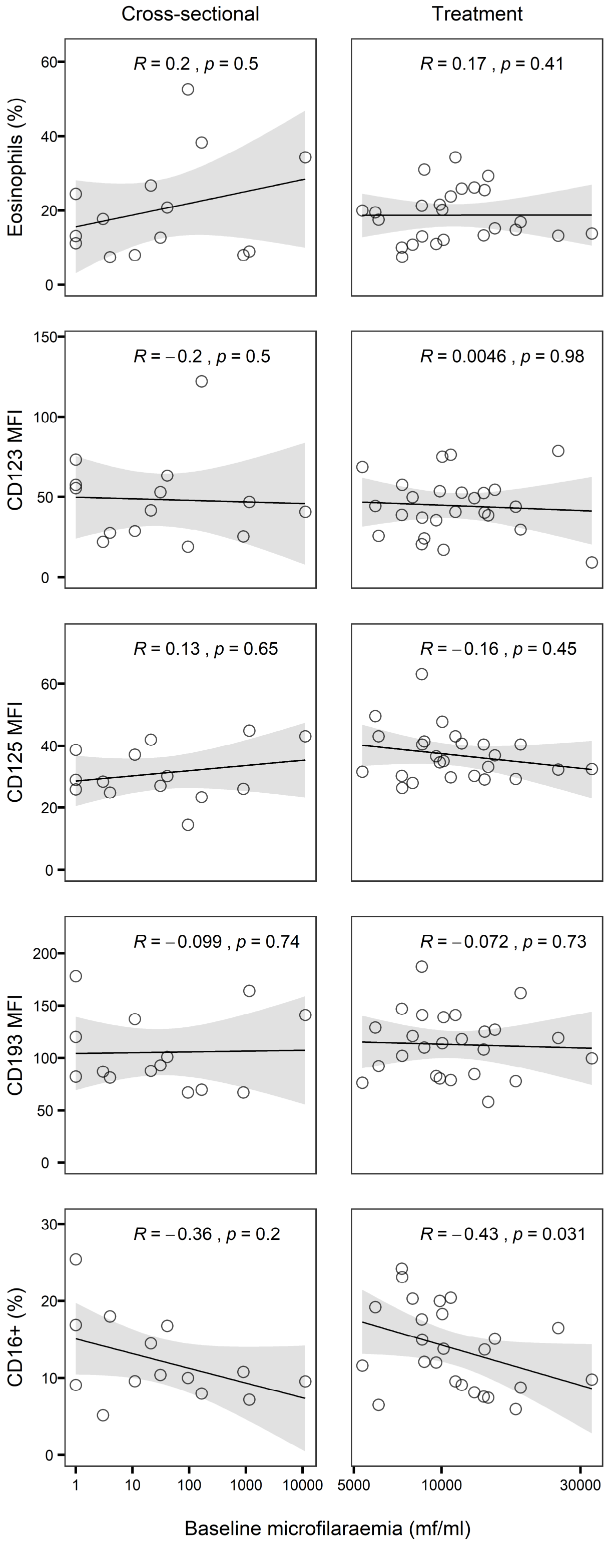

Figure C: Correlation of eosinophil activation and baseline microfilaraemia

Correlation of percentage of SSC^{hi} CD125+ CD193+ eosinophils among single leukocytes and expression of surface activation markers as median fluorescence intensity (MFI) or percentage positive and baseline *Loa loa*-microfilaraemia in the cross-sectional and treatment study. Black lines and shaded areas represent linear regression lines with 95 % confidence bands. R and p values were obtained by Spearman correlation analysis.

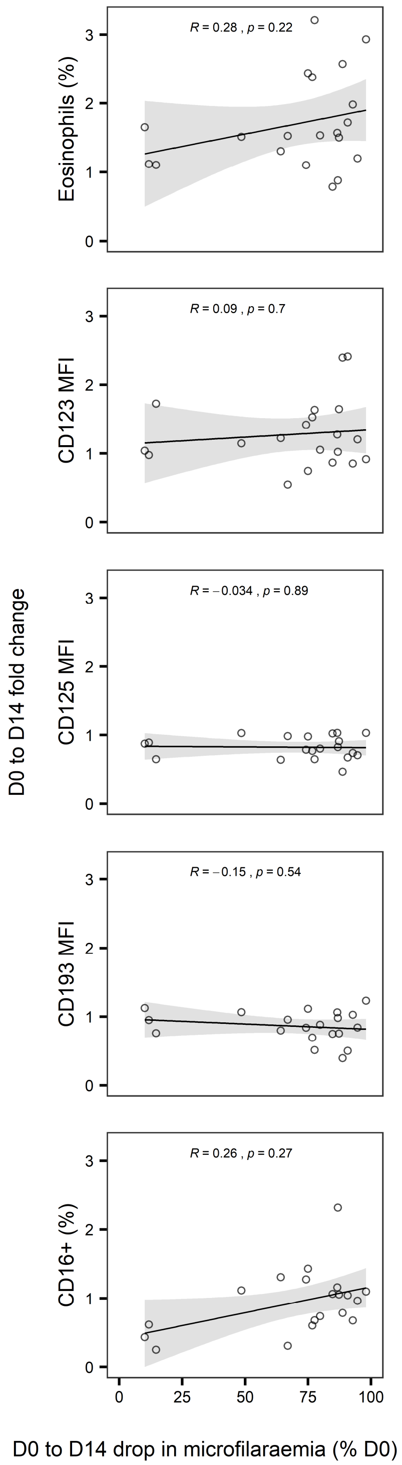

Figure D: Correlation of eosinophil activation and drop in microfilaraemia upon treatment

Correlation of day 0 to day 14 fold change of percentage of SSC^{hi} CD125+ CD193+ eosinophils among single leukocytes and expression of surface activation markers as median fluorescence intensity (MFI) or percentage positive and day 0 to day 14 drop in *Loa loa*-microfilaraemia as percentage of day 0 microfilaraemia in 20 individuals undergoing treatment with albendazole. Black lines and shaded areas represent linear regression lines with 95 % confidence bands. R and p values were obtained by Spearman correlation analysis.

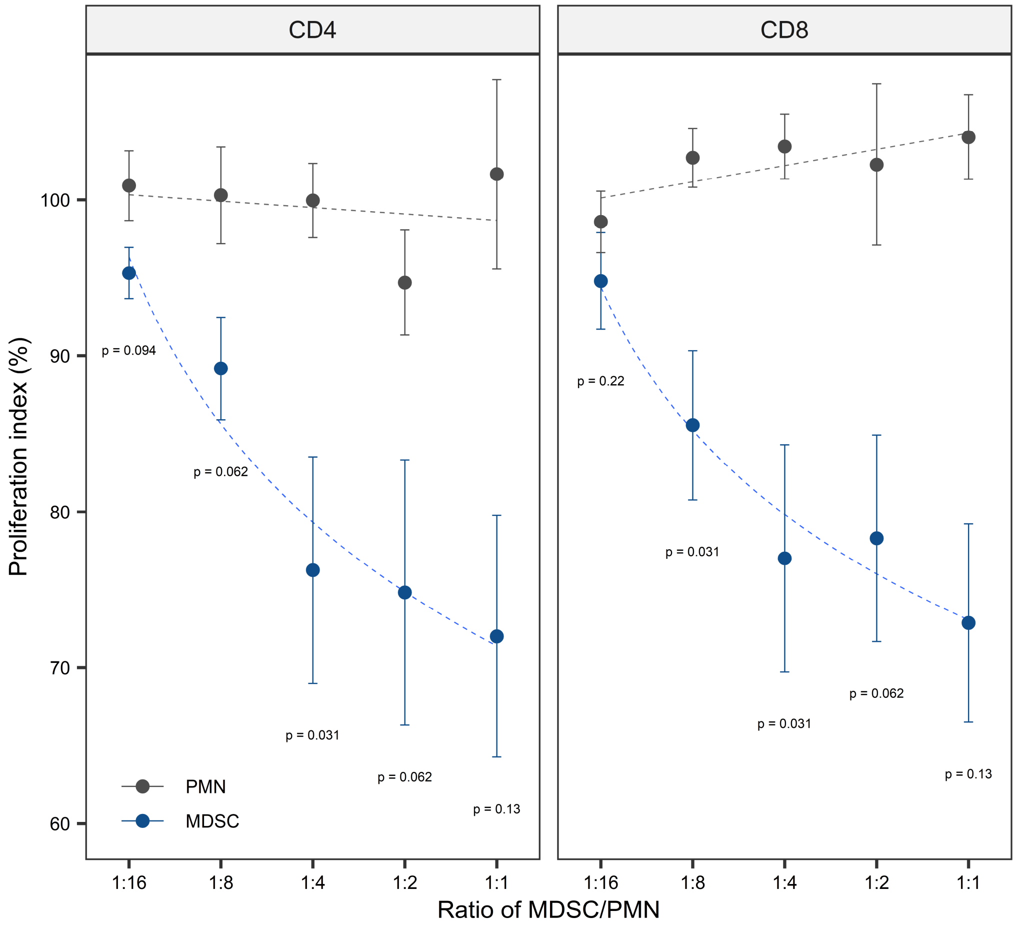

Figure E: T cell proliferation-suppression assay

Proliferation indices of CD4+ and CD8+ single live CFSE-stained healthy donor peripheral blood mononuclear cells (PBMC) after undergoing a four-day proliferation-suppression assay with increasing ratios of co-cultured polymorphonuclear myeloid-derived suppressor cells (MDSC, blue) or leukocyte controls (PMN, grey) from five study participants. Proliferation indices were normalized to the stimulated control (healthy donor PBMC only). Dots and error bars represent mean and standard error of the mean, respectively. One-sided p values were obtained by paired Wilcoxon signed-rank tests.

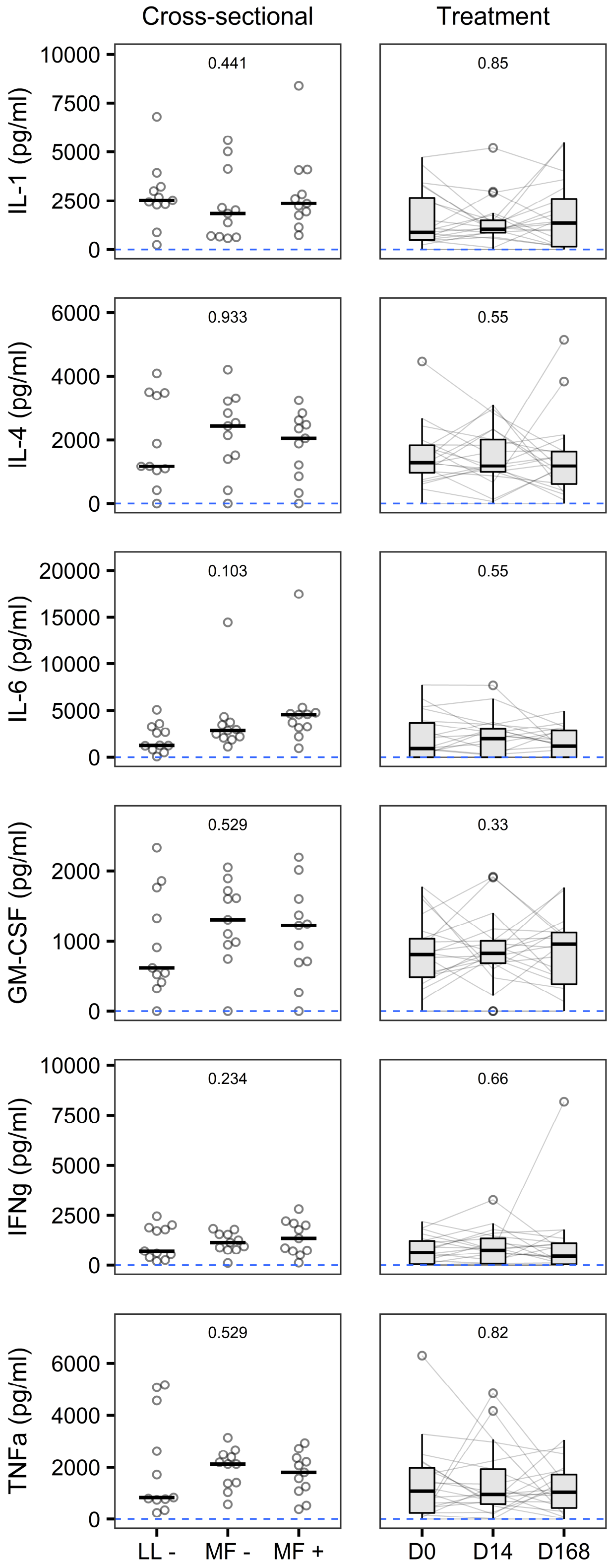

Figure F: Circulating cytokines

Plasma levels of interleukin 1 (IL-1), interleukin 4 (IL-4), interleukin 6 (IL-6), granulocyte-macrophage colony-stimulating factor (GM-CSF), interferon gamma (IFNg) and tumor necrosis factor alpha (TNFa) in age- and sex-matched *Loa loa*-uninfected (LL-), -amicrofilaraemic (MF-) and -microfilaraemic (MF+) participants in the cross-sectional study

(left column, horizontal bars represent median) and in microfilaraemic participants receiving different albendazole-based treatment regimens at baseline (D0), on day 14 of treatment (D14) and at the end of follow-up (D168) (right column, Tukey's boxplot gives median, interquartile range (IQR) and whiskers 1.5 IQR). Blue dashed lines represent lower limits of quantification (1 pg/ml). P values were obtained by Friedman's and Nemenyi's post-hoc test. Matched cytokine data were unavailable for nine participants in the cross-sectional and five participants in the treatment study.
