# Supplementary material for: Eosinophils, basophils and myeloid-derived suppressor cells in chronic Loa loa infection and its treatment in an endemic setting
Source: PLoS Negl Trop Dis. 2024 May 21;18(5):e0012203. doi: 10.1371/journal.pntd.0012203 (PMC11147522; doi:10.1371/journal.pntd.0012203)
Supplement: S2 Appendix — T cell proliferation-suppression assay. (PDF) [file pntd.0012203.s002.pdf]

Supplementary Methods

T cell proliferation-suppression assay

Participant PMN-MDSC were isolated by magnet-activated cell sorting (MACS): PBMC were consecutively incubated with anti-human CD66b FITC and anti-FITC microbeads (Miltenyi Biotec, Bergisch Gladbach, Germany) in MACS buffer (PBS supplemented with 2 % FBS, 1 mM EDTA) and were run through two consecutive MS MACS columns in an OctoMACS separator (both Miltenyi Biotec) before being resuspended in autologous medium (RPMI 1640 without phenol red (Merck, Darmstadt, Germany), supplemented with 10 % PBS/plasma supernatant from PBMC isolation). Participant leukocytes collected in autologous medium after erythrocyte lysis of whole blood (FACS lysing solution, BD Biosciences, San Jose, USA) served as PMN controls. PBMC from a healthy *L. loa*-uninfected donor were stained with carboxyfluorescein diacetate succinimidyl ester (Vybrant CFDA SE Cell Tracer Kit, Thermo Fisher Scientific, Waltham, USA) according to the manufacturer's instructions and stimulated with 150 U/ml IL-2 (R&D Systems, Minneapolis, USA) and 1.5 µg/ml anti-human CD3 (BD Biosciences). Participant PMN-MDSC or PMN controls were plated with donor PBMC at ratios of 1:16, 1:8, 1:4, 1:2 and 1:1 in a 96-well plate. After 4 days at 37 °C and 5 % CO_2 cells were harvested, washed, and stained with anti-human CD4 PE (BD Biosciences), CD8 APC (BioLegend, San Diego, USA) and propidium iodide (Thermo Fisher Scientific) before acquisition by flow cytometry. FlowJo Proliferation Modelling (BD Biosciences) was used to calculate proliferation indices for CD4+ and CD8+ single live cells respectively (Fig A in S1 Appendix).
